# “Kind and Grateful”: A Context-Sensitive Smartphone App Utilizing Inspirational Content to Promote Gratitude

**DOI:** 10.1186/s13612-016-0046-2

**Published:** 2016-07-04

**Authors:** Asma Ghandeharioun, Asaph Azaria, Sara Taylor, Rosalind W. Picard

**Affiliations:** MIT Media Lab, 75 Amherst St., 02139 Cambridge, MA USA

**Keywords:** Gratitude, Inspiration, Trigger, Randomized control trial (RCT), Mood, Valence, Arousal, Contextual cue

## Abstract

**Background:**

Previous research has shown that gratitude positively influences psychological wellbeing and physical health. Grateful people are reported to feel more optimistic and happy, to better mitigate aversive experiences, and to have stronger interpersonal bonds. Gratitude interventions have been shown to result in improved sleep, more frequent exercise and stronger cardiovascular and immune systems. These findings call for the development of technologies that would inspire gratitude. This paper presents a novel system designed toward this end.

**Methods:**

We leverage pervasive technologies to naturally embed inspiration to express gratitude in everyday life. Novel to this work, mobile sensor data is utilized to infer optimal moments for stimulating contextually relevant thankfulness and appreciation. Sporadic mood measurements are inventively obtained through the smartphone lock screen, investigating their interplay with grateful expressions. Both momentary thankful emotion and dispositional gratitude are measured. To evaluate our system, we ran two rounds of randomized control trials (RCT), including a pilot study (N = 15, 2 weeks) and a main study (N = 27, 5 weeks). Studies’ participants were provided with a newly developed smartphone app through which they were asked to express gratitude; the app displayed inspirational content to only the intervention group, while measuring contextual cues for all users.

**Results:**

In both rounds of the RCT, the intervention was associated with improved thankful behavior. Significant increase was observed in multiple facets of practicing gratitude in the intervention groups. The average frequency of practicing thankfulness increased by more than 120 %, comparing the baseline weeks with the intervention weeks of the main study. In contrast, the control group of the same study exhibited a decrease of 90 % in the frequency of thankful expressions. In the course of the study’s 5 weeks, increases in dispositional gratitude and in psychological wellbeing were also apparent. Analyzing the relation between mood and gratitude expressions, our data suggest that practicing gratitude increases the probability of going up in terms of emotional valence and down in terms of emotional arousal. The influences of inspirational content and contextual cues on promoting thankful behavior were also analyzed: We present data suggesting that the more successful times for eliciting expressions of gratitude tend to be shortly after a social experience, shortly after location change, and shortly after physical activity.

**Conclusions:**

The results support our intervention as an impactful method to promote grateful affect and behavior. Moreover, they provide insights into design and evaluation of general behavioral intervention technologies.

## Background

Kindness and gratitude are among the foundational human interactions that bring people together. Their positive effect on psychological wellbeing, social interactions and organizational productivity, has long been studied and confirmed Parks and Schueller (2014). Grateful people are reported to feel more optimistic and happy Watkins et al. (2003), to better mitigate aversive experiences Emmons and McCullough (2003), and to have stronger interpersonal bonds Algoe (2012). In fact, recent studies indicate that gratitude can also benefit physical health: Gratitude interventions have been shown to result in stronger cardiovascular and immune systems, improved sleep, and more frequent exercise Post (2005). These findings motivate the development of new ways to help people learn to embrace gratitude in daily life.

Literature suggests that practicing gratitude, mostly in controlled psychological intervention settings, has lasting effects on dispositional gratitude and psychological wellbeing Carver et al. (2003); Seligman et al. (2005). Some of the most effective interventions include keeping a gratitude journal Emmons and McCullough (2003), counting blessings daily and delivering a gratitude letter in person Lyubomirsky et al. (2011).

Recently, design and development of technologies to support wellbeing and human potential, known as positive computing Calvo and Peters (2014), has grown significantly. For example, psychologists have begun to adopt pervasive technologies to research positive affect and wellbeing Howells et al. (2016); Konrath and Yan (2015). Some use mobile technology to obtain an in-situ assessment of psychological status Dufau et al. (2011); Miller (2012). Others leverage its ubiquity to make tools such as gratitude journals and lists of kindness ideas accessible everywhere Parks et al. (2012); Acts of Kindness (2015); Butterfly Effect (2015); Day One (2015); Gratitude 365 (2015); Gratitude Journal (2015); Gratitude (2015); iDo Good Deeds (2015); Random Acts of Kindness (2015). Bao et al. Bao et al. (2013) discuss the potentials and challenges of creating context-aware mobile frameworks for large-scale psychological interventions. A few examples apply such context awareness characteristics to predict and warn of mental health emergencies Burns et al. (2011). As mentioned by Calvo and Peters (2014), creative ways of supporting expressions of gratitude can be a future research path for improving wellbeing.

To the best of our knowledge, the only work that employs pervasive technology to active gratitude intervention was recently published by Runyan et al. (2014). In this study mobile phones are used to generate reminders to count blessings and, in parallel, ask questions to measure current mood. It does not, however, use inspiration as an intervention strategy nor does it utilize contextual data to explore the timing of delivery. Our work uniquely intertwines all of these concepts.

Our approach attempts to nourish intrinsic motivations for gratitude rather than imposing a set of social incentives or extrinsic rewards. As Deci and Ryan put it “intrinsically motivated activities are ones for which there is no apparent reward except the activity itself” Deci and Ryan (1975). Toward this aim, we borrowed guidelines from self-determination theory Ryan and Deci (2000) to design our intervention. Based on this theory, *autonomy*, *competence*, and *relatedness* are key components of intrinsic motivation. First, our study utilizes inspiring content—positive images, practical “thank you” ideas, famous quotes, etc.—to attempt to cultivate a feeling of gratitude rather than direct notifications. This, keeps the users in-charge of the action and thus satisfies *autonomy*. Second, we provide users with a history of all their gratitude expressions and acts of kindness. We believe this helps them feel *competent* in doing good. Third, expressing gratitude helps people feel more connected and *related* to others Algoe (2012) and makes it easier for intrinsic motivation to flourish. Overall, we aim to protect sincerity, an attribute we believe to play a significant role in gratitude’s effectiveness both for the person who expresses gratitude and the one who receives it.

We leverage ubiquitous computing technologies, only recently available, to naturally embed such content in individuals’ everyday life. Building on contemporary behavior change models Fogg (2009), we propose a way to take into account the transitional nature of human motivation. We design contextual cues, based on mobile sensor data, and take the first steps of examining when is the right time to inspire a person to show gratitude. Additionally, we introduce a novel situated experience sampling method, recording one’s mood non-obtrusively through the smartphone’s lock screen. Traditional dispositional measurements, alongside the users’ responses to the gratitude inspiring content cues, are analyzed to evaluate our gratitude intervention.

## Methods

This section will present “Kind and Grateful,” an android smartphone app we have developed for our study, embodying both novel measuring techniques and novel methods of intervention. We begin by reviewing the gratitude assessment techniques we designed and used. Next, our intervention is described, followed by its design principles and how it elicits the users’ attention. Then, we present a novel tool developed to capture smartphone users’ current mood. Finally, we describe the protocols that our two rounds of RCT followed. As we have developed our study iteratively, each section will additionally describe the main improvements that were made and our learnings.

### Measuring Gratitude and Appreciation

Scholars identify four facets to an individual’s tendency to be grateful Froh et al. (2011). Grateful people are distinguished by experiencing gratitude more frequently throughout the day, more intensely per positive event, across a wider span of experiences and with more density (i.e. grateful to more people). Traditionally, these facets have been used to conceptualize gratitude as a dispositional trait - one’s tendency to recognize and respond with gratitude. Gratitude, however, can also be experienced as an affective state. Although momentary, state gratitude has been shown to result in reciprocal pro-social displays of kindness and build its dispositional counterpart Wood et al. (2008a, b). To account for both long-term disposition and momentary affect, our study incorporates multiple methods for measuring gratitude.

**Table 1:** Surveys administered throughout the pilot study

Survey	No. of questions	Questionnaires on study timeline														
		F	S	S	M	T	W	R	F	S	S	M	T	W	R	F
GQ-6	6	+	+	+	+	+	+	+	+	+	+	+	+	+	+	+
GRAT	44	+							+							+
Ryff’s PWB	42	+							+							+

Columns show the days of the week

We have applied widely administered self-report surveys to assess dispositional gratitude. This includes the six-item GQ-6 questionnaire McCullough et al. (2002), the 44-items GRAT questionnaire Thomas and Watkins (2003), and Ryff’s PWB questionnaire Ryff (1989). The GRAT, complements GQ-6 with additional dimensions of dispositional gratitude such as resentment and social appreciations. Ryff’s PWB questionnaire supplements our data with measurements of psychological wellbeing. In each round of the study, subjects were required to fill out these surveys daily, weekly, or at the end of each stage. Tables 1 and 2 summarize when each questionnaire was administered in each round of the study. Note that the daily GQ-6 questionnaires were removed from the main study. This stems from results of our pilot study, corroborating that the GQ-6 scores remain mostly consistent on consecutive days. The main study, therefore, measures dispositional gratitude and wellbeing, only with GRAT and Ryff’s PWB, at the beginning, middle and end of the study.

**Table 2:** Surveys administered throughout the main study

Survey	No. of questions	Questionnaires on study timeline					
		F_1_	F_2_	F_3_	F_4_	F_5_	F_6_
GRAT	44	+		+			+
Ryff’s PWB	42	+		+			+

$F_i$ shows the *i*th Friday since the start of the study

**Fig. 1:**
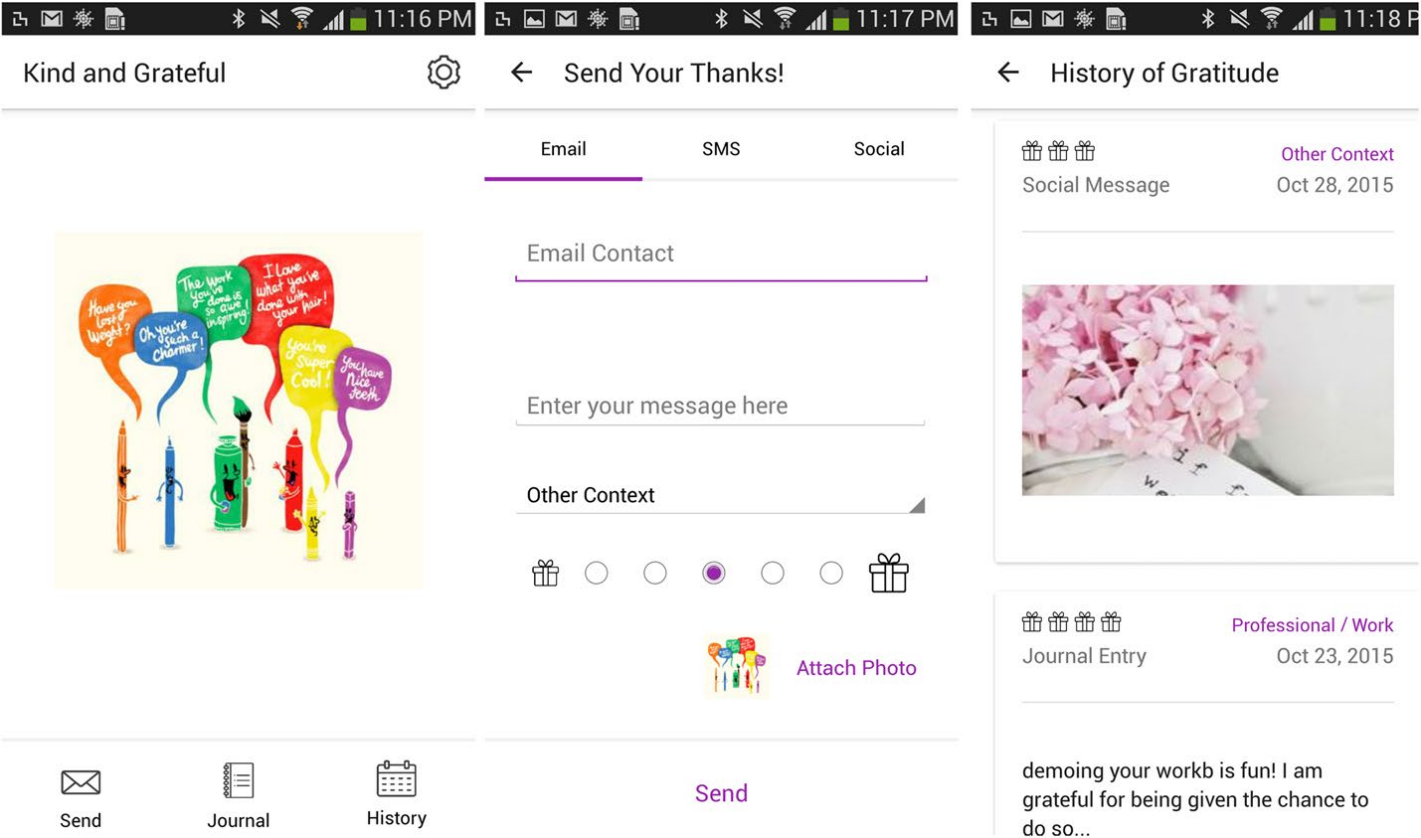
“Kind and Grateful” user interface—final version screenshots of the final version of the user interface used to express gratitude in the main study

Measuring grateful momentary affect posed an unexpected challenge. To the best of our knowledge, it has so far only been measured through gratitude interventions where participants were partaking in synthetic positive psychology exercises. This, of course, could not have been possible in the natural settings of our study. To this end, our android application, “Kind and Grateful,” was created to make it easier for people to experience and express gratitude in everyday life. Any interaction users had with the interface was logged and later analyzed. An image of the app interface is in Fig. 1.

Note that the interface does not replace, but only aggregates the various means of communications present in one’s phone. Users can thus stick to their preferred way of communications, allowing seamlessness and a wider span of expressions. Gratitude expressions were not limited to interpersonal communications. Instead, participants could also use the interface to reflect on general gratitude, say for a beautiful day, by posting content in social networks such as Facebook, Snapchat and Instagram.

**Fig. 2:**
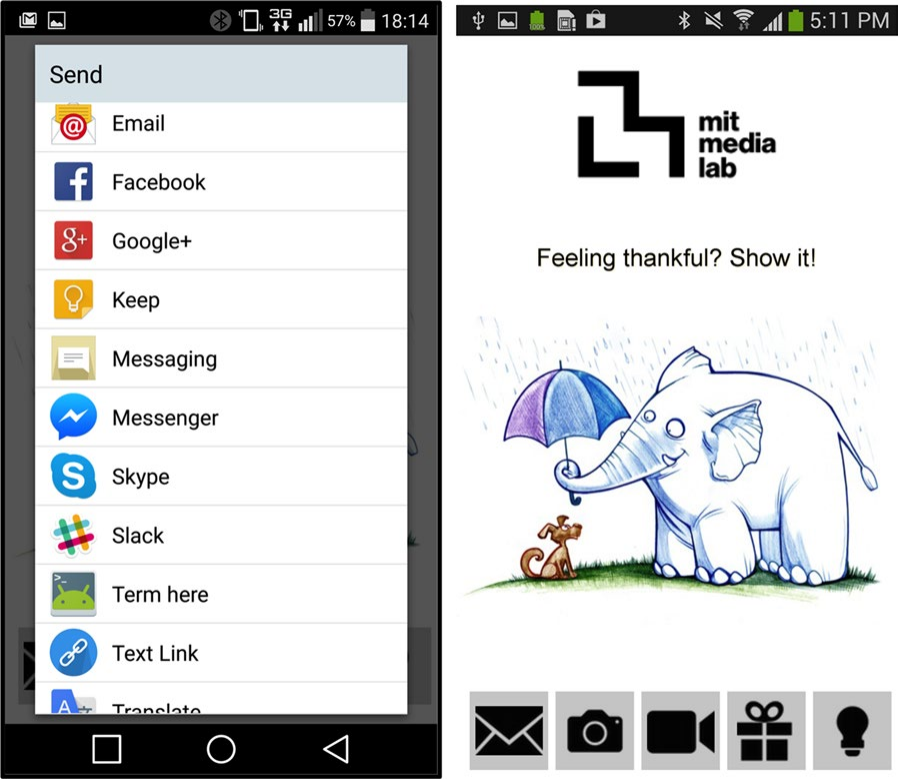
“Kind and Grateful” user interface - Original version Screenshots of the original version of the user interface used to express gratitude in the pilot study

The app interface was iteratively reviewed and evaluated by users and extensively redesigned, following Human Centric design principles. Many improvements, such as consolidating unused button, simplifying ranking and automating recipient search, were made targeting the interface’s usability. We would like to highlight two improvements that were introduced in the main study, following users’ feedback and requests. The first is a feature which allows sharing the inspirational content with others—either directly or affixed with a personal note. The second is a history option, allowing users to view and reflect on their past expressions of gratitude. These, we believe, allude to underlying social and retrospective elements of gratitude, driving users’ needs, which facilitate its expression. Figures 1 and 2 illustrate the app interface used in the main study and the pilot study, respectively.

We consider four facets of dispositional gratitude. This approach provides a multidimensional perspective on our observations and proposes a future framework to analyze expressions of gratitude. The four facets and their measures are:

- *Frequency* The number of time participants communicated or expressed gratitude through the interface in a given time.
- *Intensity* How grateful the person is currently feeling. Participants could rate their gratitude intensity using a 1-to-5 visual likert scale measure.
- *Span* The number of circumstances that show up in the users’ expressions of gratitude. We have predefined 11 categories, such as family, health, nature and faith, and tagged each expression with them.
- *Density* The number of different recipients to whom gratitude was expressed using our app. This number was calculated for each participant separately, daily and for the entire week. Gratitude recipients were not restricted to members of the study.

Whenever the users were using the app to express gratitude, they were automatically asked to provide indications about the expression’s intensity, span, and density. For example, the users were asked to rate, on a five star scale, how strongly they currently feel grateful to measure intensity. Likewise, Span was measured by requesting users to check relevant circumstances from the predefined list of options. Density, on the other hand, was measured automatically with the number of recipients to whom a message or a post were addressed. All of the above indicators were recorded by the app interface for analysis.

### Gratitude Intervention Design

**Fig. 3:**
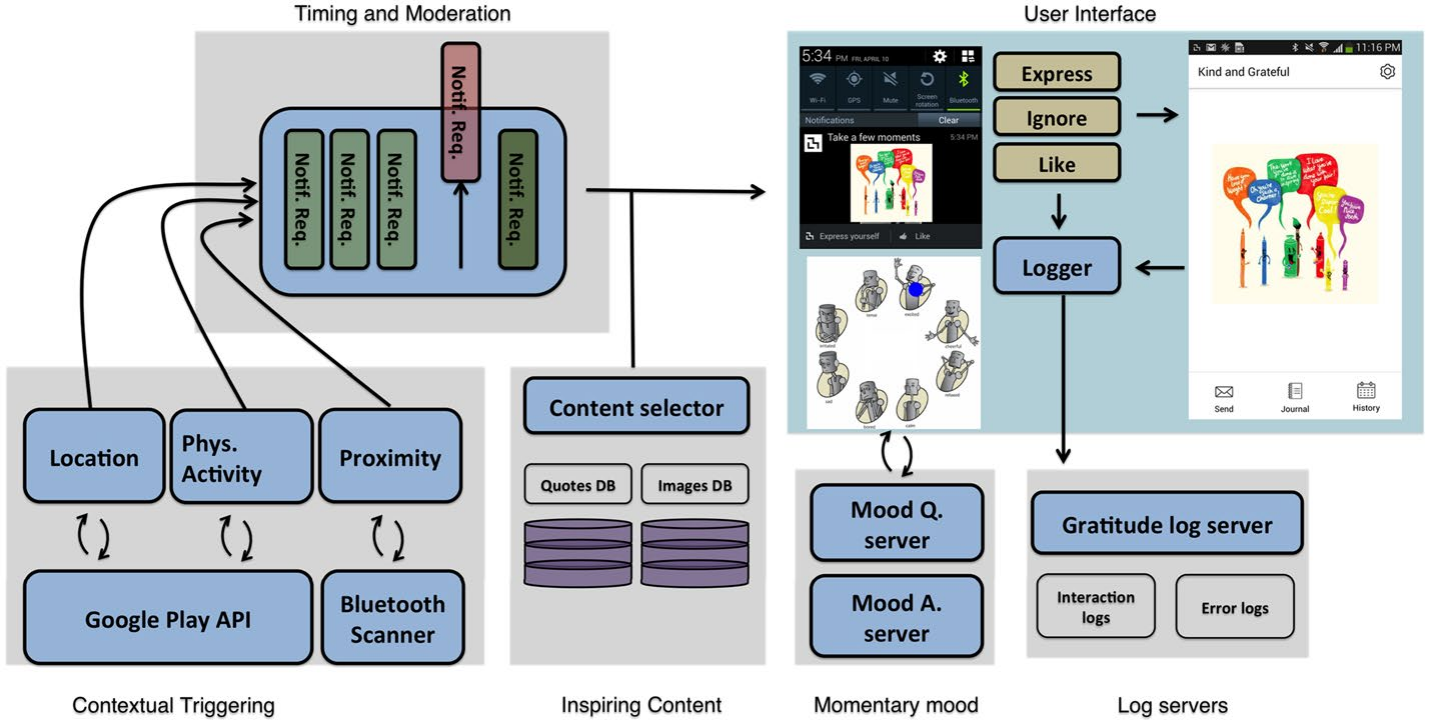
Top-level system design technical overview of the system’s components and operation

“Kind and Grateful” provides users with gratitude inspiring content—oth images and textual prompts—packaged as phone notifications. Figure 3 illustrates the system components and app’s flow of operation.

Notifications were generated either periodically (every 2 h) or by requests from contextual triggers. Three contextual triggers were implemented in an attempt to characterize when is the right time to prompt the users with inspiring content. Each trigger represents a different hypothesis: (1) Location changes: A notification is requested whenever users move from one region, where they have spent at least half an hour, to another (region here is a circle with <400 m radius). (2) Social proximity: Proximity to other group members is inferred by constantly monitoring the phone’s Bluetooth interface. A notification is requested when peers that have been close to each other depart. (3) Physical activity: Physical activity is estimated via the phone’s inertial sensors. When the user is active for over 7 min, a notification is requested. We consider active as either walking, running or cycling estimated with over 75 % confidence.

**Fig. 4:**
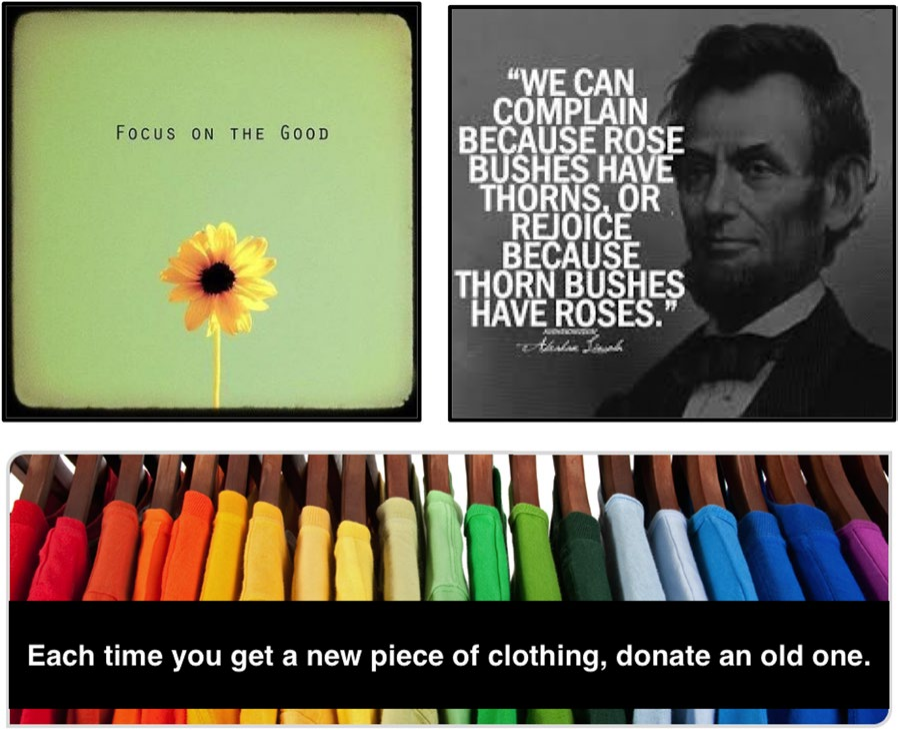
Sample inspirational content Screenshots of sample inspirational content. Content range from practical ideas for kind and thankful acts, universal idioms, quotes and advice related to gratitude, or general advice toward positivity

To avoid over-loading, our system implements a queuing mechanism, moderating the frequency of notifications to no more than once every 30 min. To maintain contextual relevance, notification requests that have not been fulfilled for a long period of time expire. Once a notification is generated, content is randomly selected from a database containing over 250 inspiring items. The items are arranged in two categories with similar selection likelihood. The first category contains practical ideas for kind and thankful acts. The second category comprises universal idioms, quotes and advice related to gratitude. Figure 4 exemplifies some of the provided content and the different categories. Within the same category, a balanced ratio of textual and visual content was preserved.

An important modification was introduced to the social proximity trigger, differentiating between the pilot and main studies. In the pilot study the participants were recruited in groups representing their social circles. Accordingly, social proximity logging and triggering was implicitly limited to the study participants of the same group. In the main study, however, the app was modified to allow users to explicitly specify the members of their social circle, whether they participate in the study or not. Members of the social circle were properly informed, and consented by providing their Bluetooth address for monitoring. This not only allowed scaling the number of recruited participants, but also provided users with the flexibility to better represent their social circle.

### User Interface and Engagement

Aspiring to make gratitude habitual, we have designed our intervention to naturally be embedded in everyday life. We package inspiring content as phone notifications, tapping to the frequent awareness users give to their mobile phones. These notifications invite the user’s attention in exactly the same way emails and instant messages do. They show up both on the user’s phone screen and wearable devices. In particular, Samsung Gear Live smart-watches were provided to some groups, to evaluate the efficiency of pervasive wearable technologies in the settings of such positive psychology interventions.

**Fig. 5:**
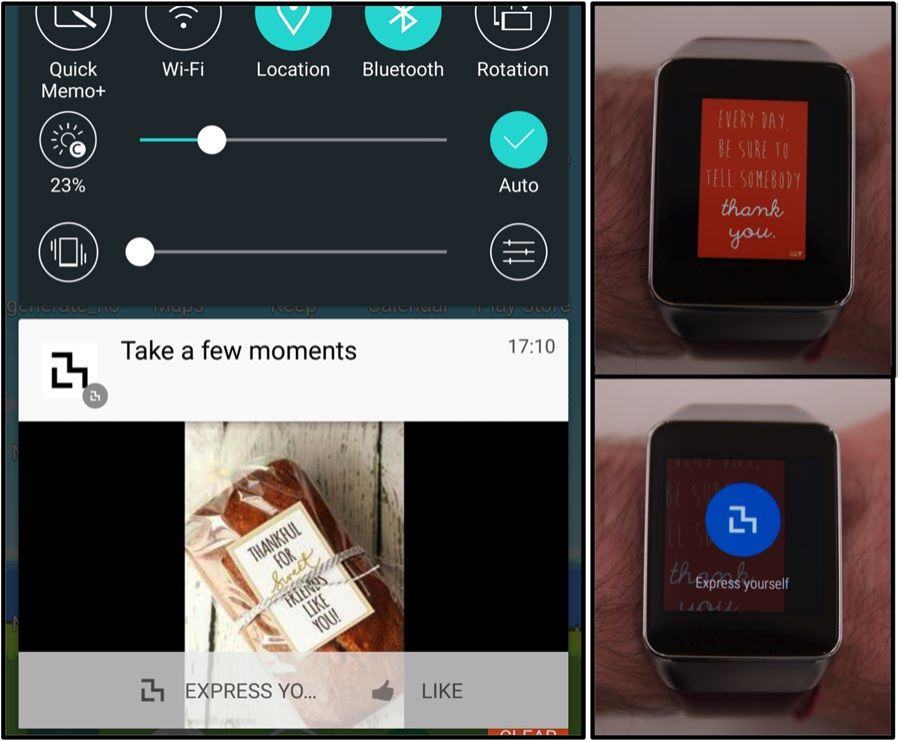
Gratitude notifications notifications appearing on the smartphone and smart-watch

Once a notification is generated, the inspiring content can be viewed in the phone’s task bar, as shown in Fig. 5. Three responses by the user are possible: (1) Ignoring the notification by swiping it out of the task bar; (2) Using the “Like” button to signify that the notification’s content or timing was appreciated; or, (3) Launching the mobile interface and expressing gratitude through it by pressing the “Express” button. The content and possible actions are all accessible via the smart-watch, as well as the phone. Figure 5 shows how the notifications appear on each device.

**Fig. 6:**
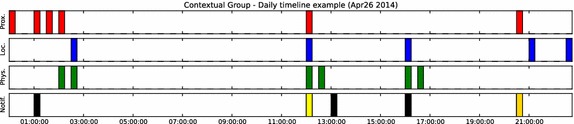
A typical intervention day [User C4 April 26 2015]. The* top rows* illustrates notification requests from the contextual triggers. The* bottom row* illustrates the generated notifications and how the user interacted with them. Each* color* represents an action;* Black*: Ignored;* Yellow*: Like;* Gold*: Gratitude Expression

Figure 6 exemplifies how a typical intervention day was experienced by one of our users. The first three rows illustrate notification requests generated by each of the contextual triggers. The bottom row illustrates the notifications that were delivered to the user, and how he interacted with each of them.

### Sampling Current Mood

Transient mood has been shown to affect judgments and behaviors in numerous circumstances Forgas and Moylan (1987). Accordingly, we hypothesized that the users’ mood may affect their sense of gratitude and their willingness to express it. Thus, sampling mood periodically adds a potentially useful source of information to study expressions of gratitude, to cancel out the effect of mood on sense of gratitude, or to find the interconnection between the two. Though having this source of data is very helpful, it can be costly. Even using the most recent experience sampling methods for asking a person to rate his/her mood eight times a day in a long run, can be annoying and time-consuming. However, we tackle this problem and minimize the burden of experience sampling on the user by building upon the already adopted habits. Smartphone users unlock their phones 10 to 200 times per day Truong et al. (2014). By embedding “micro-questions” in the unlock screen, we can collect self-reported information unobtrusively. These questions can be answered by a single swipe gesture within less than a second. Consequently, we don’t interrupt users to ask them how they are feeling. Instead, they report their mood optionally as a side product of unlocking their phone screens.

**Fig. 7:**
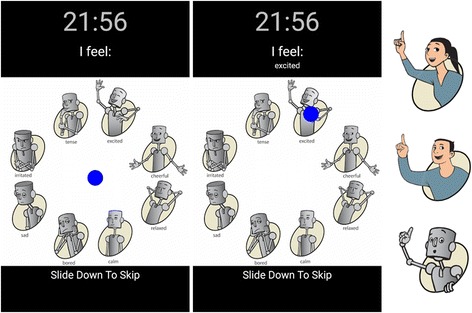
“Mood Tracker” interface screenshots of the mood unlocking screen. To unlock their phone, users swipe in the direction of the their mood. Three types of icons were available. Participant could choose their favorite icon set (female, male, robot)

Toward this end, we have developed an android application that gathers self-reported mood data from the lock screen of an android phone which we call “Mood Tracker”. For our application, we prompted users to finish the statement “I feel:” by dragging a blue circle to one of eight different moods displayed by a character before unlocking the phone. The prompt appeared on the user’s lock screen eight times a day, about once every two hours starting as early as 9 a.m. (depending on the user’s unlocking behavior). When the prompt wasn’t displayed, the user just swipes the screen to unlock the phone. The user also had the option to skip the prompt and respond later if the timing of the question was inconvenient. Figure 7 shows the user interface of the app that overlays the phone lock screen. Participants can choose their favorite set of icons from the three options: female, male, and robot. The displayed characters are based on “Pick-A-Mood” (PAM), a cartoon based mood-reporting instrument Desmet et al. (2012) in which each icon resembles a different mood. PAM characters are structured according to the two-factor model of mood Watson and Tellegen (1985), one of the most prevalent mood assessment strategies in self-reported mood measurements. This model considers two dominant dimensions for mood: valence (pleasure–displeasure) and arousal (high energy–low energy). Horizontal and vertical axes in PAM correspond to valence and arousal respectively. In other terms, characters are sorted such that their valence increases (becomes more positive) by going to the right and their arousal increases by going up.

### Study Protocol

Assessing the effectiveness of our intervention, we conducted two rounds of user studies, following a randomized control trial protocol. To quantify the changes in appreciation and gratitude, we compared our intervention data to data collected from independent Control groups and from a non-intervention reference stage. The non-intervention reference stage removes bias induced by factors such as personality traits, cultural backgrounds, and social norms that may differently characterize our groups. The Control groups allow canceling shifts in subjects’ gratitude that stem from environmental and temporal factors Cunningham (1979) like extreme weather, major sport events, national holidays, and tragic events in the news.

#### Pilot Study

Fifteen (N = 15) MIT students participated in our pilot study for a period of 2 weeks. To capture social interactions and proximity, all participants were recruited in groups. Each group represented a close social circle where members interact with each other on a daily basis. Participants aged 18–30 included 10 undergraduates and five graduate students. Seven participants were female, and the rest were male. Each group was randomly assigned to one of three conditions: Control, Periodic, and Contextual. During the first stage of the study (first full week), all the groups only gained access to the measuring interface of our mobile application through which they have all been asked to express gratitude.

During the second stage (second full week), the Periodic group was additionally notified with inspiring content periodically, every 2 h, while the Contextual group was notified with inspiring content according to the contextual cues that were previously detailed. The Control group continued to use the app as in the first week.

Three groups—one for each intervention condition—were provided with Samsung Gear Live smart-watches. Two additional groups were recruited a week later and were randomly assigned to Control and Contextual conditions. These additional groups were not provided with smart-watches, but had access to the remaining parts of our system. We used these groups to assess how wrist notifications influenced the delivery of our intervention (see Table 3 for summary).

**Table 3:** Summary of participating groups

Round	Group	Intervention	Smartwatch	No. of participants
Pilot	I	Control	+	2
	II	Contextual	+	4
	III	Periodic	+	3
	IV	Control	−	3
	V	Contextual	−	3
Main	VI	Control	−	14
	VII	Contextual	−	13

Participants were randomly assigned to an intervention condition

#### Main Study

A second round of the study was conducted a couple of months later, after analyzing and learning from the results of the pilot study. This round lasted 5 weeks with (N = 27) participants, including nine MIT undergraduates, 14 graduate students, and four staff members. 14 participants were females and the rest were males; they aged between 18 and 35.

Based on the results from the pilot study, we focused the main study on the impact of the contextual triggers. Both qualitative and quantitative data from the pilot study showed participants in the periodic group were more likely to ignore notifications when they happened at specific times comparing to when they were context-sensitive. Consequently, this round included only Contextual and Control conditions, and did not include the Periodic condition. Participants were randomly assigned one of the two conditions as summarized in Table 3. Like before, during the first stage of the study (this time 2 weeks), both groups only gained access to the measuring interface of our mobile application. During the second stage (3 weeks), the Contextual group received inspiring content according to the contextual cues, while the Control group kept using the app as in the first 2 weeks.

As previously mentioned, a set of improvements were made in the mobile app, differentiating the two rounds of the studies. We base these improvements on implicit (user behavior analysis) and explicit (comments) feedback from the pilot users. These included: (1) Removing the daily GQ-6 questionnaire, (2) Changes in the interface through which gratitude is expressed, (3) Flexible selection of one’s monitored social circle, and (4) Situated mood measurement through the smartphone’s lock screen. Aside from those, a central data collection server was implemented. The server collected the app’s logs from all the users in real time via the Internet. It allowed not only to scale the number of users participating in the study, but also to avoid face-to-face meetings between the participants and the study personnel, minimizing their interference.

Due to ambiguous reactions of participants to the smart-watches and scaling considerations, we removed the watches from the second round of the study and maintained only the smartphone app interface.

## Results and Discussion

This section presents quantitative results and qualitative observations obtained from our RCTs. We first assess the impact of our intervention, followed by an analysis of the role of transient mood in gratitude expressions. Then, we examine the relevance of our contextual cues. We conclude by discussing users’ reactions to the inspirational content.

### Intervention Impact

To fully capture the impact of our intervention, we analyze our results from two perspectives: daily and stage-wise. The daily perspective explores changes within or between consecutive days, whereas the stage-wise perspective compares the intervention and reference periods. For each perspective we present measurements of mood, momentary gratitude and dispositional gratitude, and relate those to the relevant intervention data from the mobile app.

#### Zooming In: Daily Changes in Gratitude Measures

**Fig. 8:**
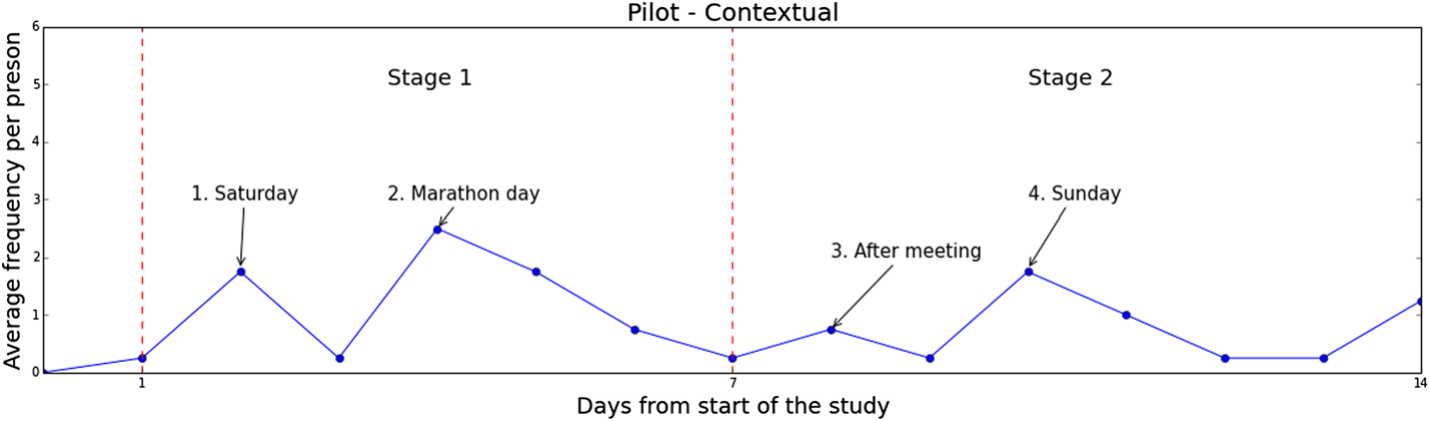
Main study—daily changes in practicing gratitude daily changes in practicing gratitude over the course of 2 weeks exemplified with data collected from the contextual group from the main study

**Fig. 9:**
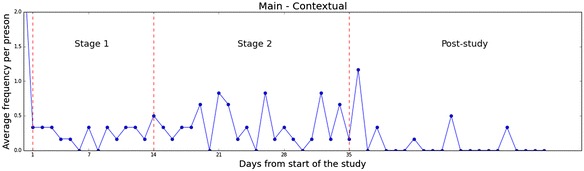
Pilot study—daily changes in practicing gratitude daily changes in practicing gratitude over the course of 2 weeks exemplified with data collected from the contextual group from the pilot study

Figures 8 and 9 illustrate, on a daily timeline, the changes of gratitude expression frequency, for the pilot and main studies respectively. The presented frequency values are averaged per person within the corresponding condition group. For the sake of brevity, we visualize the results collected from the Contextual groups and demonstrate only the frequency facet of gratitude expressions; our observations, however, apply to the rest of the groups and facets just as well.

This timeline visualization reveals patterns, hinting at factors that impact grateful experiences and expressions. First, we can see the influence of temporal landmarks Dai et al. (2014) (holidays and special events) in our data. An increase in gratitude expressions is noticed around weekends, arrows “1” and “3” in Fig. 9. Moreover, the rise in gratitude expressions marked by arrow “2” accords well with the Boston Marathon holiday. The event seems to have a lasting effect which only gradually wears off.

Second, we notice the presence of social influences in the results of the pilot study. Throughout this study, we have held face-to-face meetings with our participants, to collect data locally stored on their phones. We see a slight increase in all measurements around these dates (arrow “4”). We attribute such increases to a sense of commitment that our meetings might have triggered in our participants. System improvements in the main study, have successfully removed such bias, as the data from the main study suggest.

We have also analyzed the daily changes GQ-6 scores. As expected, the scores remain fairly steady throughout our study. We anticipate dispositional gratitude changes to become apparent only in longer intervention periods. For reference, a 9 weeks intervention period was necessary in Emmons and McCullough (2003) to start noticing effects in dispositional gratitude.

For the most part, an overall decrease in gratitude expressions is observed during consecutive days of the pilot study. We associate this with the novelty effect of introducing our new technology and note that this effect wears off as time goes by, becoming less apparent during the second week. Extending the main study to five weeks has successfully reduced the significance of the novelty effect in our results. Clearly, changes in gratitude expressions in the main study do not follow a similar trend. In fact, data from the main study show that more than half of the participants in the Contextual group continued using our system longer than the study’s period. Such a behavior is unlikely to stem from a novelty effect. Though insufficient to suggest adoption, this finding encouragingly supports the successful non-intrusive design of our intervention.

#### Zooming Out: Stage-Wise Changes in Gratitude Measures

**Fig. 10:**
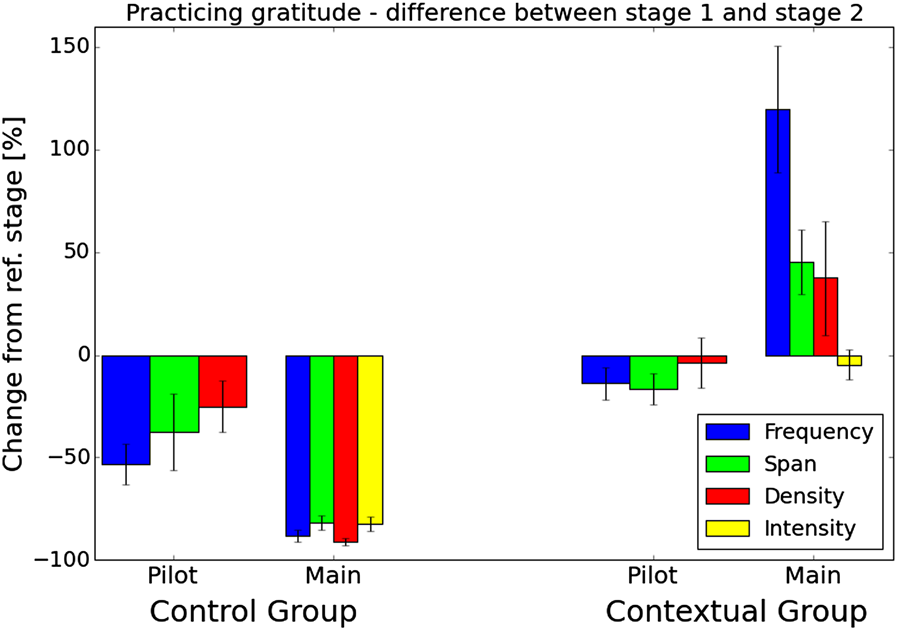
Overall changes in practicing gratitude measurements of practicing gratitude. Percentage of changes in different aspects of gratitude between stage I (1 week for pilot users, 2 weeks for main users) and stage II (1 week for pilot users, 3 weeks for main users)

Next, we examined changes in gratitude expressions from a stage-wise perspective. We calculated the percentage of change in each momentary gratitude facet between the reference and intervention stages of the studies. Figure 10 shows the results of both the pilot and the main studies. We obtained group values by averaging the change among the group members. The error bars in the illustrated results represent the standard deviation. The blue, green, red, and yellow bars stand for frequency, span, density, and intensity respectively.

The results from the main study reveal the significance of our intervention. A comparative look between the groups uncovers its effectiveness. Clearly the Contextual group expressed gratitude more frequently (p = 0.003), across wider span (p = 0.002), more densely (p = 0.031), and intensely (p = 0.002). For example, the frequency of practicing gratitude increased by more than 120 % on average in the Contextual group. In contrast, a decrease of 90 % was obtained in the frequency of thankful expressions in the Control group. Similar trends are evident in all facets of gratitude measurements. They support our intervention as an impactful method for changing gratitude related behaviors. We attribute the negative changes between stages in the pilot study to the aforementioned novelty effect.

**Fig. 11:**
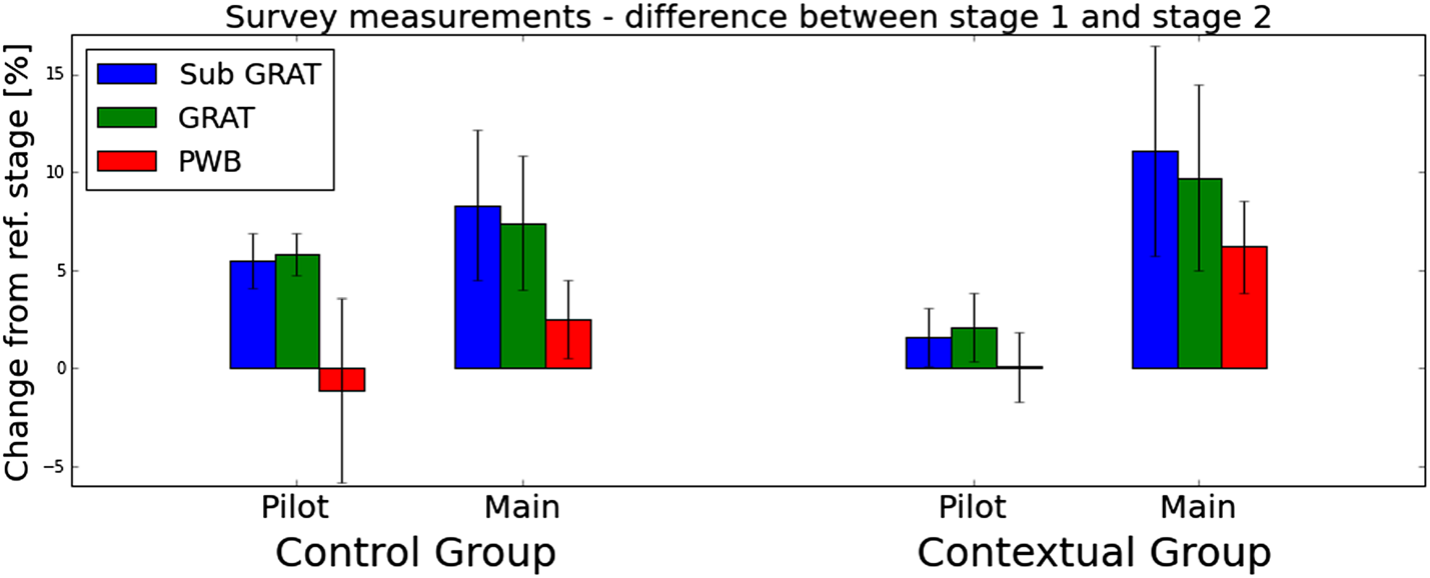
Stage-wise percentage changes in dispositional gratitude measures stage-wise percentage changes in measures of dispositional gratitude across different groups in the pilot and main studies. Each* bar* shows the average change per group and the* error bars* show one standard deviation

We also analyzed the change in dispositional gratitude, measured by the GRAT and PWB scores. To account for the multicultural background of our participants, we dropped two of the questions from the GRAT questionnaire which relate to Thanksgiving and Christmas. However, no significant differences were observed between the full and reduced GRAT scores. Figure 11 shows the results from both the pilot and the main study. The changes in the pilot study are subtle, coinciding with our previous assumption regarding the longitudinal nature of dispositional gratitude. In the main study, however, after the course of five weeks, slight improvements are observed in the different measures. The main study’s GRAT results indicate that the intervention groups appear to be improving more than the Control group; This difference, however, is not statistically significant. We speculate that a longer period will be required for these improvements to show statistical significance.

To supplement our quantitative results, we conducted in-depth post-experiment interviews with our participants. These shed light on individuals’ experiences and support our findings about the intervention’s effectiveness. Many participants reported our system to have made them more aware of everyday kindness. F1 said: “As I was writing down what I was grateful for, I became more and more thankful! I began to see things I haven’t seen before.” Some mentioned positively how the inspiring content was embedded in their everyday routine. D2 reported: “I remember a time, anxiously looking at my watch, when a notification showed up and made me smile.” Some participants reported the app helped them to reappraise the situation even when faced with difficulties. 242C said: “I used the journal feature to write down things that I felt grateful for. Sometimes I had a bad day but still tried to find good in it.”

### Mood and Gratitude Interplay

**Fig. 12:**
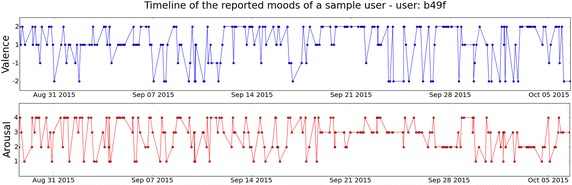
Daily mood fluctuations daily mood oscillations of a sample user (user: b49f)

Inspired by previous literature, we investigated the interplay between momentary gratitude and mood, in the context of our intervention. We exemplify in Fig. 12 mood samples from a single participant, obtained by our novel tool. As expected, these exhibit frequent oscillations daily and even within a single day. To simplify our analysis, we first project the eight possible mood descriptors to a single affective dimension—either valence or arousal. Then we collapse the samples to binary values (positive or negative valence, high or low arousal). As a result, “Irritated”, for instance, will be coded as negative valence and positive arousal.

To capture current mood more reliably, we dropped mood reports that took less than a second to answer. We base this approach on an analysis from a previous pilot deployment of our experience sampling technique. There, we observed that users who spent less than a second answering lock-screen questions, were likely to arbitrarily select an answer only to gain access to their phone. After removing invalid data points, we partitioned the mood data into blocks of two hours and calculated the most frequent mood in each block (if any). We looked at consecutive blocks and coded a mood transition. We logged 931 transitions. Twenty one of which coincided with at least one gratitude expression.

**Table 4:** The transition probability matrix for different levels of valence when no gratitude is expressed

		V$_{t+1}$	
		Neg.	Pos.
V$_t$	Neg.	0.53	0.47
	Pos.	0.10	0.90

V$_t$ and V$_{t+1}$ correspond to the current mood and next mood respectively. Neg. and Pos. correspond to negative and positive valence. Number of data points: 910

**Table 5:** The transition probability matrix for different levels of valence when gratitude is expressed

		V$_{t+1}$	
		Neg.	Pos.
V$_t$	Neg.	0.20	0.80
	Pos.	0.06	0.94

V$_t$ and V$_{t+1}$ correspond to the current mood and next mood respectively. Neg. and Pos. correspond to negative and positive valence. Number of data points: 21

**Table 6:** The transition probability matrix for different levels of arousal when no gratitude is expressed

		A$_{t+1}$	
		Low	High
A$_t$	Low	0.84	0.16
	High	0.33	0.67

A$_t$ and A$_{t+1}$ correspond to the current mood and next mood respectively. Number of data points: 910

**Table 7:** The transition probability matrix for different levels of arousal when gratitude is expressed

		A$_{t+1}$	
		Low	High
A$_t$	Low	0.82	0.18
	High	0.80	0.20

A$_t$ and A$_{t+1}$ correspond to the current mood and next mood respectively. Number of data points: 21

Tables 4, 5, 6 and 7 summarize the interplay between mood transitions and gratitude expressions. Comparing Tables 4 and 5, we observe that expressing gratitude increases by 70 % the probability of transitioning from a negative to a positive valence. This suggests that gratitude expressions may increase valence. Comparing Tables 6 and 7, we note that practicing gratitude in a high arousal state increases the conditional probability of transitioning to a low arousal state by 140 %. However, no corresponding differences are observed for transitioning inversely. This suggests that gratitude expressions may have a calming effect and result in a lower arousal level. Due to the relatively small number of data points, however, longer data collecting is needed to establish the significance of these relationships.

### Contextual Relevance

Next, we assessed the relevance of our contextual triggers with respect to practicing gratitude. We base our assessment on quantitative analysis of temporal dependency and qualitative analysis of post-experiment surveys. Note that during the reference stage and in the Control group, our system still collected contextual data. Thus, it gives us insight into the context when gratitude is practiced not only with our intervention, but also without it.

**Fig. 13:**
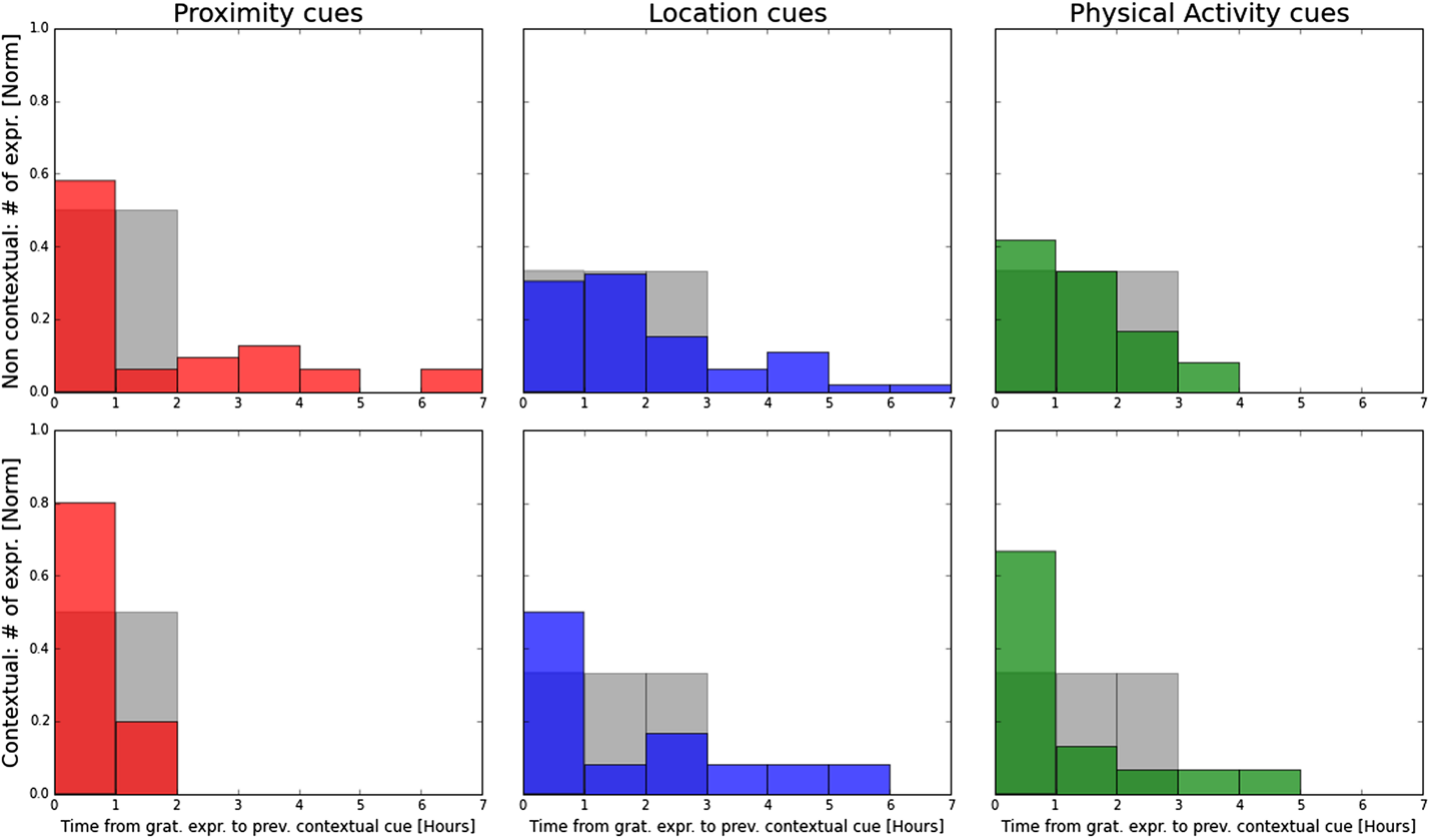
Main study—contextual relevance Temporal dependency between practicing gratitude and the contextual cues.* Top* non-intervention data from the control group and baseline weeks during which cues were computed, but not shown to the participants.* Bottom* Intervention data from the contextual group during which participants received contextual triggers.* Grey histograms* represent the null-hypothesis as a reference

**Fig. 14:**
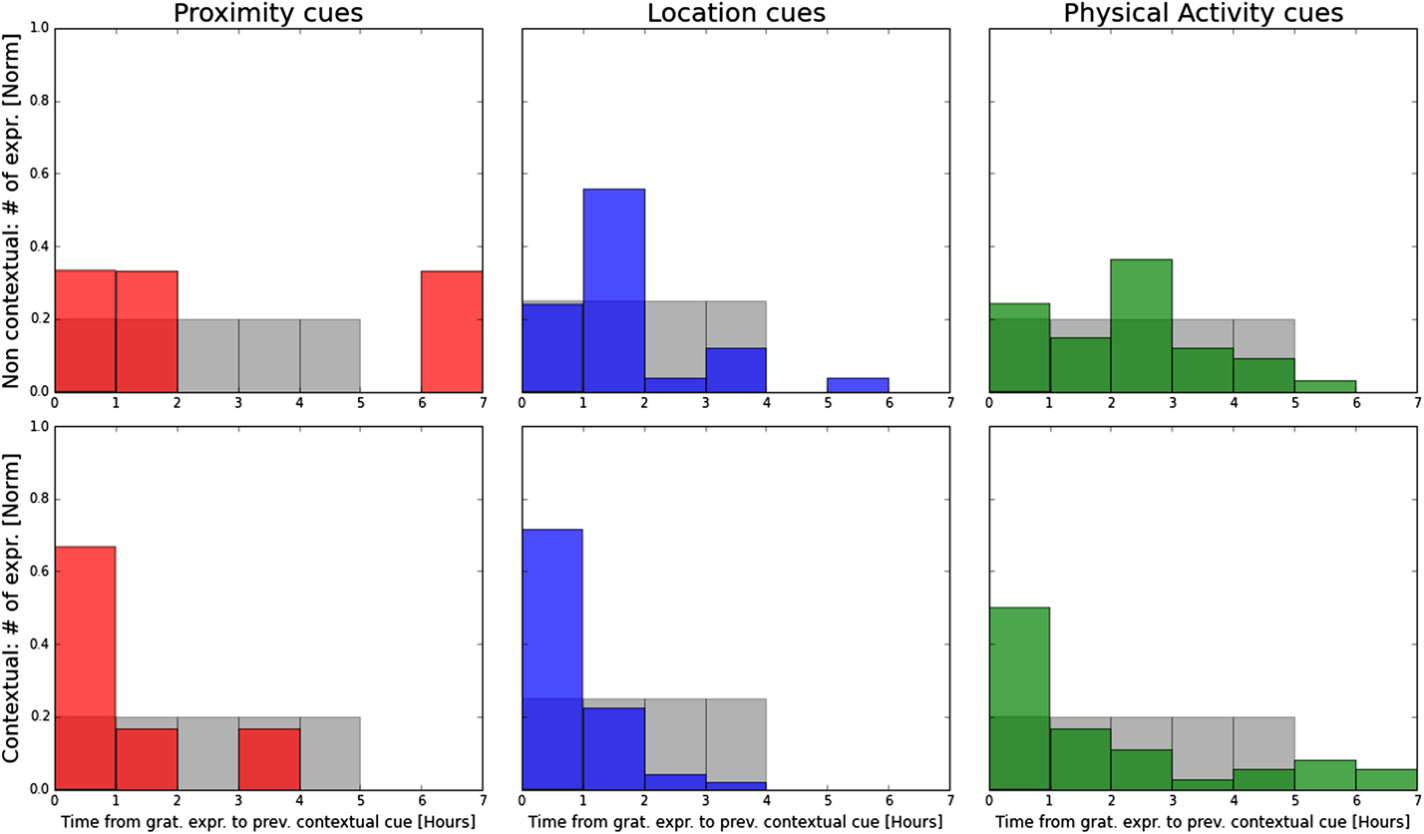
Pilot study—contextual relevance temporal dependency between practicing gratitude and the contextual cues.* Top* non-intervention data from the control group and baseline weeks during which cues were computed, but not shown to the participants.* Bottom* Intervention data from the contextual group during which participants received contextual triggers.* Grey histograms* represent the null-hypothesis as a reference

First, we demonstrate a measurement of the temporal dependency by taking each expression of gratitude individually and looking back to compute how long beforehand a contextual cue occurred. Figures 13 and 14 present the distribution histogram of these time intervals for the pilot and main studies respectively. Our results juxtapose intervention data collected from the Contextual group with non-intervention data gathered from the reference weeks and the control group.

To evaluate our results, we must compare them to the null hypothesis - i.e. the histograms that would have been generated if practicing gratitude were completely independent from our contextual cues. Such histograms are illustrated in Figs. 13 and 14 in grey. They take the distribution of the contextual cues throughout the active hours of a typical day, and calculate the expected value of the time intervals, assuming that gratitude expressions were random.

For the non-intervention data, our results show that gratitude expressions are more likely to happen an hour after social proximity or physical activity than stochastically. This finding supports our hypothesis on the contextual nature of practicing gratitude and bolsters the design guidelines of our intervention.

The contextual intervention data indicate an even stronger tie between the contextual cues and practicing gratitude. This can be reasoned either by the effect of our intervention or by the relevance of our cues to when the intervention should be delivered. More specifically, the plots show most of the gratitude expressions happened up to an hour after social proximity trigger (80 % of the time in the pilot study and about 70 % in the main study). A similar trend is observed in the location and physical activity triggers. In fact, tying the intervention with the location cues produced the highest increase in the likelihood to express gratitude shortly after.

Interviews with our participants have exemplified situations where the context of our intervention highly contributed to its effect. Participants indicated times, specifically while commuting at the end of the day, when the contextual notifications were highly effective. They associated their efficiency with being unoccupied and mentally in need for some positivity after a long day. C4 reported: “I was commuting on the train when I suddenly received a notification suggesting I would give my seat to someone else. Surprised by its relevance, I immediately cleared my seat to an elder woman standing nearby.” Many participants have also expressed particular satisfaction with the activity and social proximity triggers. A9C7 mentioned, when leaving a social event “[...] I just had a notification from the app which reminded me about helping someone :)”, so he decided to return and help clean-up the place.

### Inspirational Content

To evaluate the role of inspiring content, we analyzed the users’ interactions with the notifications and gathered their feedback through interviews and surveys. No significant trend was observed in effectiveness of the content category (practical kindness vs. universal gratitude advice) or type (visual vs. text). Also, individual preferences varied among participants. Some found the content very influential while others reported being moved by only specific quotes and images. Most of the participants tended to judge the content according to how it related to their personal life, rather than its practicality. This implies that our categorization does not accurately represent variations in individual preferences and personalizing the content might increase its effectiveness.

## General Discussion

Though targeting gratitude behaviors, our study teaches valuable lessons that can easily be applied to the design of general Behavioral Intervention Technologies (BIT) and contribute to the field of positive computing Calvo and Peters (2014). We discuss these in this section, covering principles that can be applied to BIT system design, and practices useful for BIT system evaluation.

### BIT Design Principles

First and foremost, our study underscores the role of contextual cues in triggering behavior change. Our daily surroundings are instrumented with many sensors providing rich contextual and behavioral data. These can be leveraged to piece together an image of the users’ current state, constructing higher level events, such as settling down in a new location, physical activity, commute hours, and social activity. BIT designers can build on such events to design just-in-time delivery of behavioral interventions. Our study exemplifies how three such triggering schemes are constructed and evaluated for promoting gratitude. Similarly different schemes can be designed for other interventions.

We present significant improvements in our intervention efficiency, optimizing only for the intervention delivery times. We attempt to find such times by hypothesizing on general contexts in which the users will either have higher motivation or will be more willing to take the effort to engage in the target behavior. Our work highlights social interactions as a powerful context for intervention delivery. We observed that recruiting a socially connected group with our new intervention significantly increased its effectiveness. User interviews suggest that personalizing the triggering scheme can even further strengthen the intervention’s efficiency. We consider adopting such an approach in future work.

Finally, we note an important interplay between the users’ current mood and our behavior intervention. Sometimes, a simple behavior, like showing gratitude, can help transition from negative to positive moods. Reminding users about the prospects and triggering them at the right affective state may improve the positive affect component of wellbeing more effectively. We present a novel technique which enables mood sampling in natural settings. Such a tool can be broadly applied to many BIT designs. It can be used not only to assess the intervention’s effects on one’s affective state, but also as an input to design smart mood-aware intervention delivery.

### BIT Evaluation

Our set of RCTs emphasizes some experimental design principles for evaluating BITs. These are not necessarily conceptually novel; however, our work highlights their importance when evaluating BITs in natural settings. We note the importance of including both a baseline period and a control condition in the experimental design. The former addresses bias from individual differences, while the latter accounts for temporal landmarks such as holidays, weekends and special events. Using both of these factors helps provide control in natural settings.

Special attention should be given to the novelty effect, when designing new BITs. The first few hours, days, weeks, or months interacting with a new BIT might be very different from how the user would interact with the system in the long run. We present an iterative approach in which the duration of the novelty effect is assessed in a pilot study, and then accounted for in a second study. The second study is designed to be long enough to allow the novelty effect to wear off. We also recommend designing the BIT to involve minimal interaction with the study’s investigators. As seen in our pilot study, frequent meetings with the participants may contribute to their using it more (perhaps from desire to please the experimenters) than they would when left on their own. When the tool is built for long-term individually-driven use, the best test of its success is with long-term individually-driven use.

## Conclusions

The two rounds of the RCT support our intervention as an impactful method to promote thankful behavior and grateful affect. It resulted in improvements of dispositional gratitude (GRAT) and psychological wellbeing (PWB) measures. Relative to the Control group, the intervention groups showed a pronounced increase in practicing gratitude. These encouraging results were demonstrated in multiple gratitude facets and in spite of the influence of powerful external factors such as the novelty effect or temporal landmarks. The strongest influence was observed in the average frequency of practicing gratitude, comparing the reference weeks with the intervention weeks of the main study. This number increased by more than 120 % in the Contextual group. However, it decreased 90 % in the Control group. Our statistics also hint at the relation between the momentary aspect of grateful affect and general mood, suggesting that gratitude expressions might increase valence and have a calming effect. We show that the notifications in the Contextual condition had higher likelihoods of culminating with thankful expressions. Our indications strongly suggest that social proximity, location changes and physical activity are relevant cues for inspiring to express gratitude. We also observed how relevant inspirational content can fuel thankful behavior.

For future work, we would like to explore alternative methods for measuring momentary gratitude, as well as improvements to our intervention. Though expressing gratitude through our mobile app provides broad information about our users’ grateful affect, it is nonetheless effort demanding. Light-weight methods, such as a single-click grateful experience report, should be introduced and experimented with our intervention. In addition, we were inspired by user feedback to implement reinforcement learning techniques in future work. Such techniques can be utilized to tune the content and context of the phone notifications according to the user’s personal preferences. They can also assist in tuning the frequency of the system’s intervention by taking into account the users’ explicit responses to them.
